# 474. Unique Treatment Challenges with Multisystem Inflammatory Syndrome in Children (MIS-C) compared to Kawasaki Disease Shock Syndrome

**DOI:** 10.1093/ofid/ofab466.673

**Published:** 2021-12-04

**Authors:** Rachel Downey Quick, Keren Hasbani, Donald Murphey, Mariosl Fernandez, Kenneth Shaffer, Rachel A Quirt, Sarmistha Bhaduri Hauger

**Affiliations:** 1 Dell Children’s Medical Group, Austin, TX; 2 Pediatric & Congenital Cardiology Associates, Dell Children’s Medical Center of Central Texas, Associate Professor at Dell Medical School at the University of Texas at Austin, Austin, Texas; 3 Dell Children’s Medical Center; University of Texas at Austin Dell Medical School, Austin, TX; 4 Dell Children’s Medical Center, University of Texas at Austin Dell Medical School, Austin, TX; 5 Pediatric & Congenital Cardiology Associates, Dell Children’s Medical Center of Central Texas, Dell Medical School at the University of Texas at Austin, Austin, Texas; 6 Dell Children’s Medical Group, Austin, Texas

## Abstract

**Background:**

Kawasaki disease (KD) and Multisystem Inflammatory Syndrome in Children (MIS-C) associated with Coronavirus Disease 2019 present similarly with mucocutaneous symptoms and fever. Both syndromes can progress to shock. Successful treatments for MIS-C are largely based on proven KD management. As more patients with MIS-C are treated, protocols are adjusted. Infectious Diseases (ID) specialists are often early consultants in these cases. Understanding differences in how body systems are affected in MIS-C versus KD is essential for management.

Figure 1. Cardiac changes among patients with Kawasaki Disease shock syndrome (KDSS) and Muti-system Inflammatory Syndrome (MIS-C)

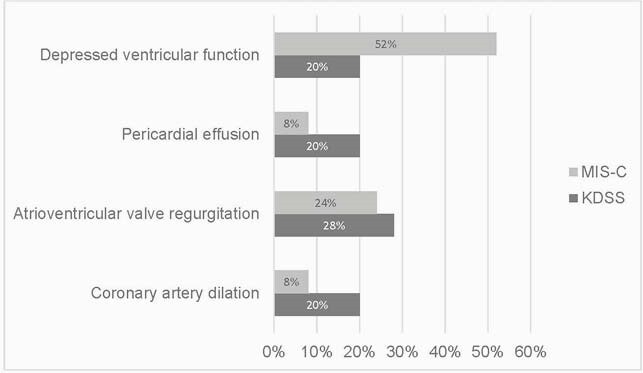

**Methods:**

This is a single hospital comparison of 25 cases of MIS-C with mucocutaneous presentation and symptoms of shock and 25 consecutive cases of KD Shock Syndrome (KDSS). Cases were compared for demographics, symptoms, cardiac abnormalities, medical treatments, and cardiac recovery.

**Results:**

Patients with MIS-C develop symptoms of shock including sustained hypotension and tachycardia at 3 times the rate of patients with KD (45% vs 13%; p< 0.001). On echocardiogram, left ventricular myocardial dysfunction, assessed by ejection fraction, is more commonly noted in cases of MIS-C than KDSS (fig 1). About half of patients with MIS-C show left ventricular myocardial dysfunction initially with normalization by 6 months post-presentation in the majority (96%).

**Conclusion:**

Cardiac changes and shock events related to KD and MIS-C are thought to be caused by differing inflammatory mediators. By comparing these two syndromes, we can determine ways to manage each optimally. MIS-C often results in left ventricular myocardial dysfunction, which is rarer in KD cases. Fluid resuscitation with multiple fluid boluses followed by inotropes to treat hypotension in cases of in MIS-C puts increased strain on the already weakened myocardium. Early intravenous immunoglobulin (IVIG) administration, even in the presence of mild hypotension, can simultaneously provide the patient with additional fluid and decrease the underlying inflammatory process. This prompt treatment might reduce the need for pressor support while protecting the myocardium from further damage. As early consultants in MIS-C, ID providers should be educated regarding the unique cardiac challenges of MIS-C and avoid delay in IVIG treatment and cardiologist and intensivist consultation.

**Disclosures:**

**All Authors**: No reported disclosures

